# Grb2 Induces Cardiorenal Syndrome Type 3: Roles of IL-6, Cardiomyocyte Bioenergetics, and Akt/mTOR Pathway

**DOI:** 10.3389/fcell.2021.630412

**Published:** 2021-03-22

**Authors:** Jin Wang, Xuefeng Sun, Xu Wang, Shaoyuan Cui, Ran Liu, Jiaona Liu, Bo Fu, Ming Gong, Conghui Wang, Yushen Shi, Qianqian Chen, Guangyan Cai, Xiangmei Chen

**Affiliations:** Department of Nephrology, Chinese PLA General Hospital, Chinese PLA Institute of Nephrology, State Key Laboratory of Kidney Diseases, National Clinical Research Center for Kidney Diseases, Beijing Key Laboratory of Kidney Diseases, Beijing, China

**Keywords:** CRS-3, GRB2, cardiomyocytes, kidney, mitochondria

## Abstract

Cardiorenal syndrome type 3 (CRS-3) is damage to the heart following acute kidney injury (AKI). Although many experiments have found that inflammation, oxidative stress, and cardiomyocyte death are involved in cardiomyocyte pathophysiological alterations during CRS-3, they lack a non-bias analysis to figure out the primary mediator of cardiac dysfunction. Herein proteomic analysis was operated in CRS-3 and growth factor receptor-bound protein 2 (Grb2) was identified as a regulator involving AKI-related myocardial damage. Increased Grb2 was associated with cardiac diastolic dysfunction and mitochondrial bioenergetics impairment; these pathological changes could be reversed through the administration of a Grb2-specific inhibitor during AKI. Molecular investigation illustrated that augmented Grb2 promoted cardiomyocyte mitochondrial metabolism disorder through inhibiting the Akt/mTOR signaling pathway. Besides that, Mouse Inflammation Array Q1 further identified IL-6 as the upstream stimulator of Grb2 upregulation after AKI. Exogenous administration of IL-6 induced cardiomyocyte damage and mitochondrial bioenergetics impairment, whereas these effects were nullified in cardiomyocytes pretreated with Grb2 inhibitor. Our results altogether identify CRS-3 to be caused by the upregulations of IL-6/Grb2 which contribute to cardiac dysfunction through inhibiting the Akt/mTOR signaling pathway and inducing cardiomyocyte mitochondrial bioenergetics impairment. This finding provides a potential target for the clinical treatment of patients with CRS-3.

## Introduction

Cardiorenal syndrome (CRS) has been defined as “disorders of the heart and kidneys whereby acute or chronic dysfunction in one organ may induce acute or chronic dysfunction of the other” (House et al., 2010). Hence, five classifications of CRS have been identified: acute cardiac dysfunction-induced acute kidney injury (CRS type 1, CRS-1), chronic cardiac abnormalities-evoked progressive chronic kidney disease (CRS type 2, CRS-2), acute renal kidney-related compromised cardiac performance (CRS type 3, CRS-3), chronic kidney loss-mediated adverse cardiovascular events (CRS type 4, CRS-4), and systemic diseases (diabetes or sepsis)-induced cardiorenal depression (CRS type 5, CRS-5) (Okamoto et al., 2019; Zununi Vahed et al., 2019). From an epidemiological point of view, CRS-1 (Atici et al., 2019; Virzi et al., 2019) has been shown to occur in ∼25% of patients hospitalized with acute decompensated heart failure, and CRS-2 (Novosel et al., 2014; Angelini et al., 2015) has been estimated to develop in 45–63% of patients with chronic heart failure. The morbidity of CRS-4 (Suresh et al., 2017; Edmonston et al., 2018) is ∼10% in patents with end-stage renal disease. Importantly, CRS-3 (Sharma et al., 2013; Clementi et al., 2015) often occurs in elderly people with acute kidney injury (AKI) requiring intensive care, and more than 70% of patients with acute kidney injury develop CRS-3. Thereby, CRS-3 is a critical challenge for physicians from a treatment standpoint.

Considering the high prevalence of CRS-3, intensive research has been initiated to explore the molecular basis of CRS-3, and thus several theoretical hypotheses have been proposed (Clementi et al., 2015; Di Lullo et al., 2017, 2019), including oxidative stress, inflammation response, hemodynamic abnormalities, and activation of the sympathetic system/renin–angiotensin–aldosterone system. Although the above-mentioned theoretical mechanisms are able to explain the pathological alterations of CRS-3, they lack a single-factor hypothesis to describe the process on how AKI triggers distant cardiomyocyte dysfunction or death.

Growth factor receptor-bound protein 2 (Grb2), a kind of adapter protein, regulates the signal transductions of epidermal growth factor receptor and mitogen-activated protein kinase (MAPK) (Ijaz et al., 2018). Following studies further elucidate its necessary role in cell proliferation, migration, invasion, development, and apoptosis through regulation of the receptor tyrosine kinase (RTK) (Chen et al., 2018). Besides that, dysregulated Grb2 has been noted in renal ischemia–reperfusion injury, and decreased Grb2 expression contributes tubular cell oxidative stress through suppressing superoxide dismutase expression (Zhao et al., 2020). In contrast, upregulated Grb2 is also a predictive biomarker for the progression of renal cell cancer due to its growth-promoting effects (Liu et al., 2019). In cultured mouse podocyte, Grb2 is activated by transforming growth factor-β and then activates renal fibrosis through upregulating the expression of adherent molecules such as integrin β1, FAK, and Src kinase (Zhang et al., 2013). These data show that Grb2 expression is altered by disparate stressful conditions and contributes to renal oxidative stress, growth/regeneration, and fibrosis. Unlike kidneys, increased Grb2 is deleterious for cardiomyocytes. Such a scenario is supported by recent studies which specifically delineate the pathological mechanism whereby Grb2 promotes cardiac hypertrophy through affecting extracellular-related protein kinase signal pathway (Ganesan et al., 2013; Xia et al., 2015). This is consistent with a previous observation that increased Gab2 induces chronic myocardial remodeling following myocardial infarction through the promotion of collagen synthesis (Sun et al., 2019). Besides that, the pro-inflammatory action of Grb2 in response to mechanical stress in the heart has been fully described (Zhao et al., 2020). The different roles played by Grb2 in heart and kidney make it a potential and promising molecule participating in the progression of CRS-3. Taken together, our study is aimed to investigate: (1) whether Grb2 is affected by AKI and consequently triggers cardiac dysfunction in CRS-3, (2) what the upstream molecular mechanism underlying Grb2-triggered cardiomyocyte damage is, and (3) what the intracellular events in response to Grb2 upregulation during AKI are.

## Materials and Methods

### Animal Studies and Histology

Eight-week-old male C57BL/6 mice were purchased from the Laboratory Animal Center, Chinese PLA General Hospital. The mice were housed in a temperature-controlled room under 12/12-h light/dark cycle and had free access to natural-ingredient food and water. CRS-3 model was established as previously described (Kelly, 2003; Sumida et al., 2015). In brief, AKI was induced through renal ischemia–reperfusion injury (IRI) through 35-min bilateral renal artery ischemia and 24- or 72-h reperfusion. To inhibit the activation of Grb2, a specific antagonist (ProbeChem, TB03, 1 mg/kg, i.p.) was used 30 min before renal IRI surgery. Heart tissues, blood, and kidney were collected after 24 or 72 h of renal IRI surgery (Kidder, 2014). Then, kidneys were immediately fixed in 4% paraformaldehyde for 24 h after IRI surgery.

### Histopathological Examination

Paraffin-fixed sections were stained with hematoxylin and eosin (H&E) or periodic acid–Schiff, and pathologic examinations were performed by interpreters who were blind to the patients’ identities. We examined pathologic characteristics including cell necrosis, loss of brush borders, cast formation, and tubular dilatation. We scored tubular injury semiquantitatively using a scale of –5, where 0, 1, 2, 3, 4, and 5 represent normal findings, ≤10, 11–25, 25–50, 50–75%, and >75%, respectively, based on previous studies (Cui et al., 2015; Wang et al., 2015). There were at least 10 random non-overlapping fields per animal for scoring.

### Echocardiographic Assessments of Cardiac Function

Echocardiographic measurements were obtained as recommended by the American Society of Echocardiography and using echocardiography (Zhou et al., 2020). Briefly, the mice were lightly anesthetized with 1% pentobarbital until the heart rate stabilized at 400–500 bpm; then, both conventional two-dimensional images and M-mode images of the heart were acquired in a parasternal short-axis view. Vevo Analysis software was used to calculate left ventricular (LV) ejection fraction (EF), and fractional shortening (FS). All assessments were performed in a blinded manner (Zhou et al., 2018c).

### Cardiomyocyte Isolation and Treatment

Primary cultures of mouse ventricular cardiomyocytes were prepared from the ventricles of treated mouse as described previously (Jin et al., 2018). The cardiomyocytes were plated on laminin-coated glass cover or culture plates and incubated with Dulbecco’s modified Eagle’s medium supplemented with 20% fetal bovine serum and antibiotics. To investigate the roles of IL-6 and Grb2 in cardiomyocyte damage and mitochondrial bioenergetics impairment, HL1 cells were pretreated with recombinant mouse growth factor receptor-bound protein 2 (Grb2), IL-6 (biogot, 10 ng/ml, 24 h).

### Mitochondrial Membrane Potential Analysis

Mitochondrial membrane potential was stained using JC-1 (5 nM, 30 min; excitation/emission 543/560) (Jin et al., 2018). Afterward, the cells underwent trypsinization, and fluorescence was assessed under fluorescence microscopy using a Zeiss LSM-5, Pascal 5 Axiovert 200 microscope, using LSM 5 3.2 image capture and analysis software, with a minimum of 10 high-magnification fields (×40) per section and three to four thin sections per group. CCCP (50 μM) and oligomycin (10 μM) for 20 min were used as positive and negative controls for the mitochondrial membrane potential (ΔΨm) measurements (Li et al., 2018).

### Analysis of the Mitochondrial Network

Cardiomyocytes were incubated for 30 min with Mitotracker Red FM (400 nM). Confocal image stacks were captured with a Zeiss LSM-5, Pascal 5 Axiovert 200 microscope, using LSM 5 3.2 image capture and analysis software and a Plan-Apochromat × 63/1.4 oil DIC objective as previously described (Zhou et al., 2020). The images were deconvolved with ImageJ. The average length of the mitochondria and the ratio of fragmented mitochondria were calculated as previously described (Zhou et al., 2018d).

### Western Blot

Cells or isolated heart tissues were washed twice in cold phosphate-buffered saline and lysed in radioimmunoprecipitation assay lysis buffer with phenylmethylsulfonyl fluoride. Protein concentrations for the whole-cell lysates were determined *via* bicinchoninic acid assay, and equal amounts of each protein sample (25–30 μg) were separated on 8–14% sodium dodecyl sulfate–polyacrylamide gel at 100 V; then, a Turbo Transfer System (Bio-Rad, United States) was used for 7 min to transfer the separated proteins to a polyvinylidene difluoride membrane, and the proteins were blocked for 1 h at room temperature. The membranes were incubated with primary antibodies overnight at 4°C, washed three times with Tris-buffered saline–Tween 20 (TBST), incubated for 1 h at room temperature with a horseradish peroxidase-conjugated secondary antibody, and washed with TBST; then, the protein bands were visualized with ECL Plus as directed by the manufacturer’s instructions and developed on film. The antibodies used in our study were as follows: p-mTOR (CST, 1:1,000, #5536S), mTOR (CST, 1:1,000, #2983), p-AKT (CST, 1:1,000, #4060S), AKT (CST, 1:1,000, #4691S), Grb2 (abcam, 1:1,000, #ab32037), t-Drp1 (CST, 1:1,000, #5391), p-Drp1 (Thermo Scientific Fisher, 1:1,000, #PA5-64821), β-actin (proteintech, 1:10,000, #66009-1-Ig), and GAPDH (proteintech, 1:10,000, #66004-1-Ig).

### ELISA and ATP Detection

We used a blood collection tube to take samples of whole blood and let them stand at room temperature. The remaining steps were the same as those for the detection of BNP (RayBio, United States, EIAM-BNP), Troponin T (Signalway Antibody, United States, EK3212), and IL-6 (Jonln, China, JL20268) (Zhou et al., 2018b). ATP production was determined through the Enhanced ATP Assay Kit (Beyotime, China, Cat. No: S0027) according to the manufacturer’s protocol (Wang et al., 2020b).

### LC–MS/MS Analysis

Digested peptide mixtures were analyzed on an Orbitrap Fusion Lumos (Thermo Fisher Scientific) mass spectrometer interfaced with an Easy-nLC 1000 nanoflow liquid chromatography system (Thermo Fisher Scientific) with nano-spray ionization in positive ion polarity. Samples were dissolved with 50 μl of solvent A (0.1% formic acid in water), and 5 μl was loaded to a homemade trap column (100 μm × 2 cm) packed with C18 reverse-phase resin (particle size, 3 μm; pore size, 120 Å; SunChrom, United States) at a maximum pressure of 280 bar with 12 μl of solvent A and then separated on a 150 μm × 15 cm silica microcolumn (homemade, particle size, 1.9 μm; pore size, 120 Å; SunChrom, United States) with a gradient of 7–32% mobile phase B (100% acetonitrile and 0.1% formic acid) at a flow rate of 600 nl/min for 60 min. The MS analysis was performed in a data-dependent manner with full scans (m/z 350–1,500).

### Gene Ontology and Pathway Enrichment Analysis

The potential functions of differentially expressed genes (DEGs) were analyzed by Database for Annotation, Visualization and Integrated Discovery 6.8. The Gene Ontology (GO) terms were considered to be significantly enriched when count in each term was >10 and *p* < 0.05. The related pathways of DEGs were identified using Kyoto Encyclopedia of Genes and Genomes (KEGG) and would be filtered based on the following criterion: *p* < 0.05.

### Mouse Inflammation Array Q1

We collected peripheral blood and centrifuged the specimens, storing serums at –80°C, followed by protein extraction. We measured 40 inflammation cytokines quantified by the Mouse Inflammation Array Q1 (RayBiotech, United States, QAM-INF-1) according to product instructions. A strict cutoff value is a fold change higher than 1.5 or lower than 0.67, with a *P*-value lower than 0.05. We analyzed proteomic results and visualized them with a heat map using Cluster and TreeView software.

### Statistical Analysis

Data distribution was evaluated with Shapiro–Wilk normality test. For normally distributed data, values are presented as mean ± standard error of the mean, and comparisons between two groups were performed with two-tailed Student’s *t*-test; ANOVA or repeated ANOVA and Tukey’s *post hoc* test were used for multiple comparisons or repeated measurements. Statistical analyses were performed with SPSS software (version 20.0), and *P* < 0.05 was considered statistically significant.

## Results

### CRS-3 Is Featured by Decreased Cardiac Dysfunction

**FIGURE 1:**
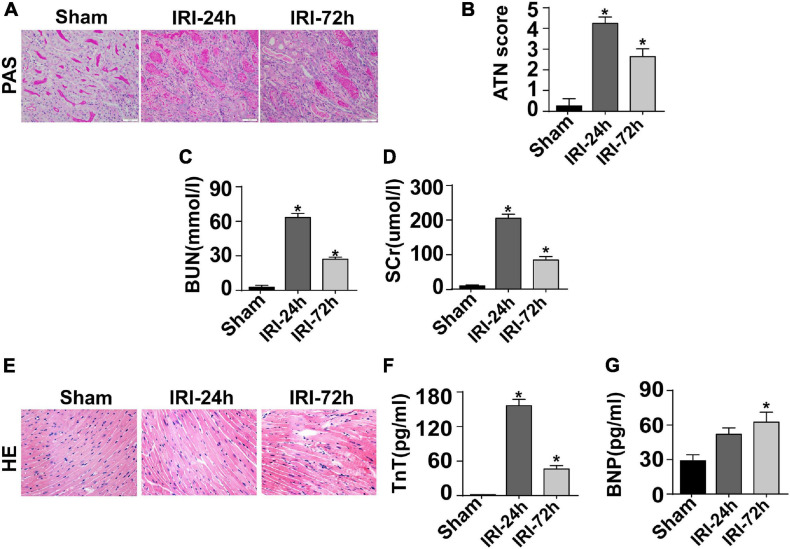
CRS-3 is featured by decreased cardiac dysfunction. **(A,B)** Periodic acid–Schiff staining for damaged renal structure. Semi-quantitative analysis of tubular injury (tubular atrophy or dilatation, loss of brush border, vacuolization, epithelial cell shedding, and denuded tubular basement membrane) was scored as follows: 0, normal; 1, <10%; 2, 10–25%; 3, 25–50%; 4, 50–75%; and 5, 75–100% of the affected area from 20 random fields. **(C,D)** Levels of BUN and serum creatinine from mice after acute kidney injury (AKI). **(E)** H&E staining for heart tissue from mice treated with AKI or not. **(F,G)** Levels of TnT and serum BNP from mice after AKI. *p < 0.05 vs. sham group.

The mouse CRS-3 model was established as previously described (Sumida et al., 2015). The upregulation of BUN and Scr (Figures 1C,D) as well as damaged renal tubules (Figures 1A,B) indicated the development of AKI. Then, blood samples and heart tissues were isolated for 24 and 72 h after AKI. There was no significant difference in the heart tissue of mice at 24 h or 72 h after renal IRI. However, the swelling of cardiomyocytes appeared at 72 h after renal IRI (Figure 1E). To evaluate cardiac damage, myocardial injury markers such as blood BNP and Troponin T were determined through ELISA. Following AKI, the levels of BNP and Troponin T were significantly elevated when compared to that in the sham group (Figures 1F,G). Besides that, cardiac contractile function was detected with the help of echocardiography analysis. As shown in Table 1, compared to the sham group, the left ventricular systolic function was not altered after AKI, whereas cardiac diastolic function, as assessed by left ventricular mass (LVM) and E/A value, was interestingly significantly impaired after AKI. These data indicate that AKI appears to primarily blunt cardiac diastolic function instead of contraction index.

**TABLE 1:** Echocardiography after renal ischemia–reperfusion injury.

	Sham	IRI-24H	IRI-72H
IVSd (mm)	1.10 ± 0.04	1.03 ± 0.07	0.95 ± 0.06
IVSs (mm)	1.30 ± 0.07	1.27 ± 0.11	1.33 ± 0.05
LVIDd (mm)	3.22 ± 0.17	3.15 ± 0.19	3.28 ± 0.21
LVIDs (mm)	2.37 ± 0.17	2.43 ± 0.11	2.41 ± 0.19
LVPWd (mm)	0.89 ± 0.04	0.83 ± 0.06	0.85 ± 0.08
LVPWs (mm)	1.17 ± 0.11	1.29 ± 0.09	1.21 ± 0.14
LVESV (μl)	22.07 ± 2.99	21.67 ± 2.37	21.93 ± 3.61
LVEDV (μl)	47.62 ± 5.08	51.70 ± 4.72	50.89 ± 6.11
LVEF (%)	58 ± 3	61 ± 5	63 ± 4
LVFS (%)	26 ± 3	31 ± 2	27 ± 3
LVM (mg)	123.41 ± 9.06	119.57 ± 10.17	157.35 ± 13.60*
E/A	1.29 ± 0.21	1.31 ± 0.10	0.89 ± 0.09*

*p < 0.05 vs. sham group.

### AKI Induces Damage to Cardiomyocyte Mitochondria

**FIGURE 2:**
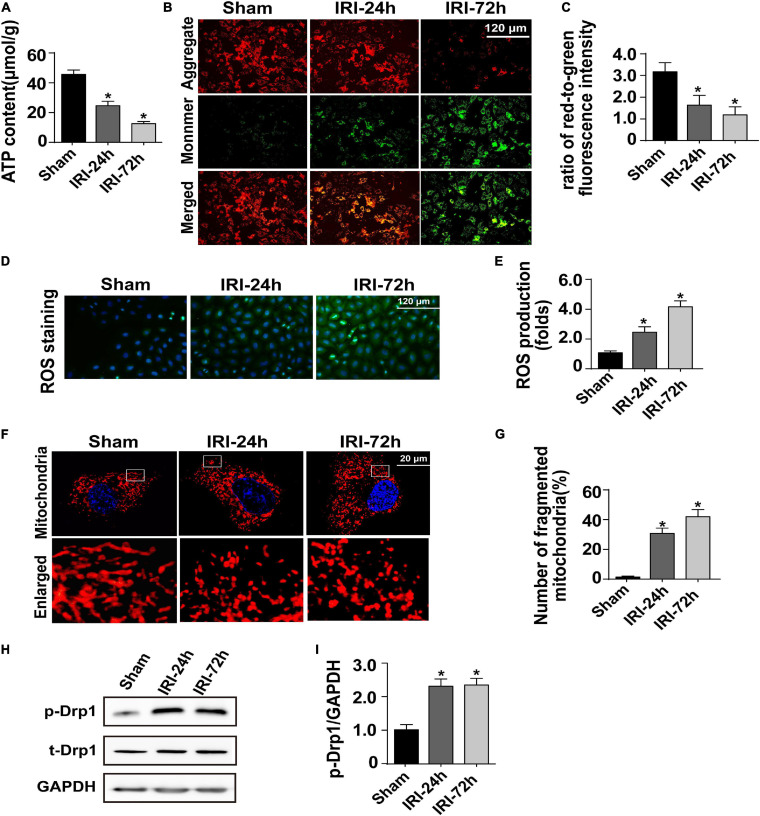
Acute kidney injury (AKI) induces damage to cardiomyocyte mitochondria. **(A)** ATP production was measured through Enhanced ATP Assay Kit. **(B,C)** Cardiomyocytes were isolated from mice after AKI. Then, mitochondrial membrane potential was determined through JC-1 probe in primary cardiomyocytes. The ratio of red-to-green fluorescence intensity was used to evaluate mitochondrial membrane potential. **(D,E)** In cardiomyocytes, DCFH-DA was used to observe the levels of intracellular reactive oxygen species. **(F,G)** Immunofluorescence of the mitochondria. The number of fragmented mitochondria in cardiomyocytes was recorded. **(H,I)** Western blots were used to analyze the expression of phosphorylated Drp1 (p-Drp1). The relative expression of p-Drp1 was evaluated in cardiomyocytes. *p < 0.05 vs. sham group.

At the molecular level, cardiac contraction/relaxation mainly controlled the content of ATP in the myocardium. Interestingly, the levels of myocardial ATP were decreased to a greater degree in mice subjected to AKI than in sham-operated control mice, suggesting that cardiomyocyte ATP metabolism is disrupted as a result of AKI (Figure 2A). Cardiomyocyte ATP production is mainly generated by mitochondria which metabolize glucose into ATP, carbon dioxide, and water, a process that is called oxidative phosphorylation.

Given the indispensable role of mitochondria in regulating myocardial ATP supply, we question whether AKI is followed by a disruption of mitochondrial function. To achieve this aim, cardiomyocytes were freshly isolated from AKI-operated or sham mice. Mitochondrial membrane potential reduction is an early sign of cardiomyocyte metabolism disturbance. Thereby, JC-1 probe was applied to stain mitochondrial membrane potential in cardiomyocytes. Compared to that in cardiomyocytes isolated from sham mice, mitochondrial membrane potential was progressively repressed in cardiomyocytes isolated from AKI-operated mice (Figures 2B,C). Besides that, as a by-product of mitochondrial metabolism, the content of intracellular reactive oxygen species, as assessed by DCFH-DA probe, was drastically increased in cardiomyocytes isolated from AKI-treated mice when compared to that in cardiomyocytes from the sham group (Figures 2D,E).

Based on recent studies, mitochondrial morphology shift acts upstream of mitochondrial metabolism disruption. Excessive mitochondrial fragmentation formation promotes the dissipation of mitochondrial membrane potential, resulting into impaired oxidative phosphorylation as well as decreased ATP production. With the assistance of immunofluorescence using a mitochondria-specific probe, we observed a cleavage of mitochondria from an elongated network into small spheres or short rods in cardiomyocytes isolated from AKI-treated mice (Figures 2F,G). This morphological shift may be a result of increased mitochondrial fission because western blot analysis demonstrated that Drp1 phosphorylation, a classical marker of mitochondrial division, was significantly elevated in cardiomyocytes isolated from AKI-treated heart (Figures 2H,I). Taken together, our results confirmed that mitochondrial metabolism damage as well as mitochondrial morphology disturbance would be the intracellular molecular events underlying AKI-mediated myocardial damage.

### Myocardial Grb2 Expression Is Increased Following AKI

**FIGURE 3:**
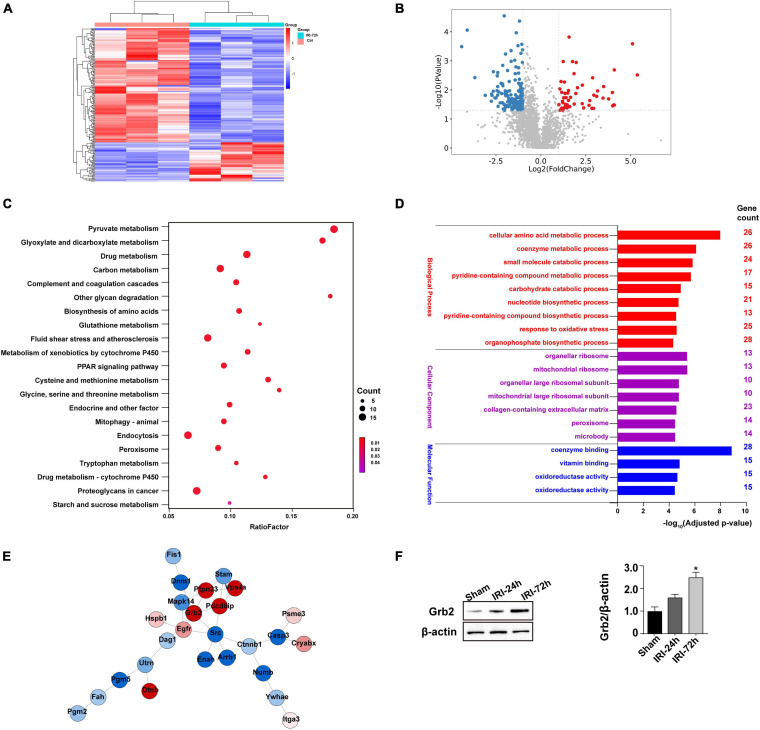
Myocardial Grb2 expression is increased following acute kidney injury (AKI). **(A)** Heat map of the altered genes in the heart tissue after AKI. **(B)** VolcanoPlot of the altered genes in the heart tissue after AKI. **(C)** The Kyoto Encyclopedia of Genes and Genomes pathway analysis of altered genes in heart tissue after AKI. **(D)** Gene Ontology annotation was used to identify the molecular function, cellular components, and biological process of differentially expressed genes. **(E)** The protein–protein interaction network among the differentially expressed genes in heart tissue. **(F)** Verification experiments were performed through western blots using proteins isolated from heart tissue after AKI. *p < 0.05 vs. sham group.

To better clarify the molecular mechanism that is activated by AKI and participates into acute myocardial damage, we performed proteomic analysis through label-free technology to understand without bias protein expression in the heart following AKI. The results demonstrated that 448 proteins (*p* < 0.05, log2 fold change) were upregulated or downregulated in heart tissue (Figures 3A,B). KEGG analysis demonstrated that pyruvate metabolism, glyoxylate and dicarboxylate metabolism, starch and sucrose metabolism, and biosynthesis of amino acids were altered in the heart after AKI (Figure 3C). GO cellular component enrichment analysis demonstrated that 23 of the 448 proteins were enriched to mitochondria (Figure 3D). A biological process description of GO further illustrated that 91 of the 448 proteins were involved in intracellular metabolic events (such as cellular amino acid metabolic process, oxidative stress, small molecular catabolic process, and coenzyme metabolism process) (Figure 3D). These data suggest that cardiomyocyte metabolism or mitochondrial bioenergetics may be affected by AKI. With the help of STRING protein–protein interaction network analysis, we found that Grb2 is involved in the cross-talk and signal integration of various molecular events in the heart following AKI (Figure 3E). In fact, previous studies have also reported the key role played by Grb2 in regulating cancer metabolism and heart fibrosis (Zhang et al., 2003; Sun et al., 2019). Given these observations, we propose that Grb2 would be a potential pathological factor involving AKI-related myocardial damage. Western blots further showed that Grb2 was slightly increased 24 h after AKI and reached to statistical difference 72 h after AKI (Figure 3F). Taken together, myocardial Grb2 expression is significantly elevated by AKI, and this alteration may be a potential molecular mechanism underlying AKI-related myocardial damage.

### Exogenous Administration of Grb2 Inhibitor Improves Cardiac Performance, Cardiomyocyte Metabolism, and Mitochondrial Function Following AKI Surgery

**FIGURE 4:**
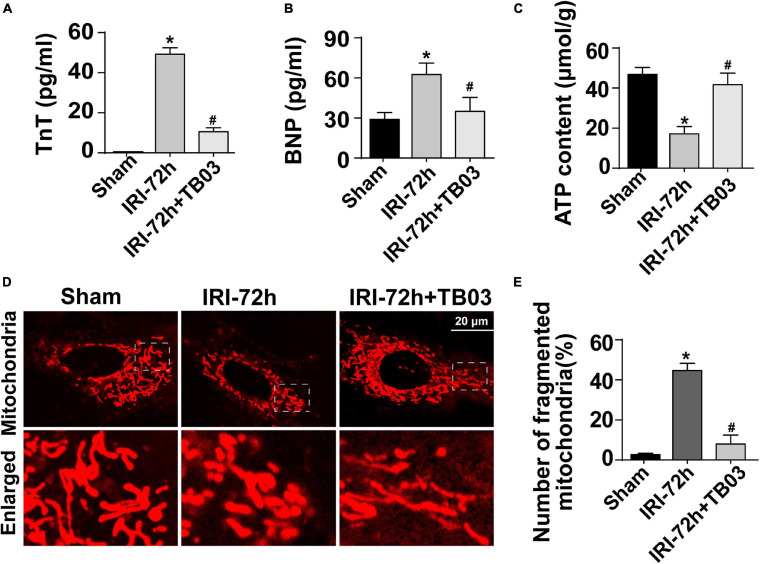
Exogenous administration of Grb2 inhibitor improves cardiac performance, cardiomyocyte metabolism, and mitochondrial function following acute kidney injury (AKI) surgery. **(A,B)** A specific antagonist of Grb2 (TB03) was injected into mice 30 min before renal ischemia–reperfusion surgery. Then, levels of TnT and serum BNP from mice after AKI were determined through ELISA. **(C)** Levels of ATP production were measured through Enhanced ATP Assay Kit. **(D,E)** Cardiomyocytes were isolated from mice after AKI. Then, immunofluorescence was used to observe mitochondrial fragmentation. The number of fragmented mitochondria was observed in response to TB03 treatment. *p < 0.05 vs. sham group, ^#^*p* < 0.05 vs. IRI-72h group.

To delineate the pathogenic mechanisms by which increased Grb2 promotes cardiac dysfunction, a specific antagonist of Grb2 (TB03) was injected into mice 30 min before renal ischemia–reperfusion surgery to fully block the activation of Grb2 during or after AKI. Besides that, Grb2 was mainly upregulated 72 h after AKI, and therefore this time point was used in the following functional assays. In AKI-operated mice, supplementation of TB03 largely reduced the levels of Troponin T and BNP (Figures 4A,B), suggesting that Grb2 inhibition was associated with cardioprotection after AKI. Besides that, left ventricular diastolic function, which was impaired by AKI, could be normalized by TB03 as evidenced by increased LVM and E/A value (Table 2). As expected, total ATP, which was repressed by AKI, could be increased to near-normal levels once TB03 was injected (Figure 4C).

**TABLE 2:** Echocardiography after renal ischemia–reperfusion injury.

	Sham	IRI-72H	IRI-72H + TB-03
IVSd (mm)	1.10 ± 0.04	0.95 ± 0.06	0.97 ± 0.09
IVSs (mm)	1.30 ± 0.07	1.33 ± 0.05	1.24 ± 0.10
LVIDd (mm)	3.22 ± 0.17	3.28 ± 0.21	3.31 ± 0.13
LVIDs (mm)	2.37 ± 0.17	2.41 ± 0.19	2.29 ± 0.15
LVPWd (mm)	0.89 ± 0.04	0.85 ± 0.08	0.81 ± 0.10
LVPWs (mm)	1.17 ± 0.11	1.21 ± 0.14	1.25 ± 0.12
LVESV (μl)	22.07 ± 2.99	21.93 ± 3.61	23.27 ± 2.83
LVEDV (μl)	47.62 ± 5.08	50.89 ± 6.11	48.35 ± 3.20
LVEF (%)	58 ± 3	63 ± 4	60 ± 2
LVFS (%)	26 ± 3	27 ± 3	28 ± 2
LVM (mg)	123.41 ± 9.06	157.35 ± 13.60*	127.42 ± 8.37^#^
E/A	1.29 ± 0.21	0.89 ± 0.09*	1.15 ± 0.22^#^

*p < 0.05 vs. sham group, ^#^p < 0.05 vs. IRI-72h group.

Cardiomyocytes were also isolated *in vitro* from TB03-injected mice to observe mitochondrial function. AKI-mediated cardiomyocyte mitochondria cleavage or division could be reversed by TB03 (Figures 4D,E), suggesting that cardiomyocyte mitochondrial morphology could be normalized by Grb2 inhibition during AKI. Overall, these verification experiments uncover AKI-mediated cardiac dysfunction; cardiomyocyte metabolism disruption and mitochondrial dysfunction are induced by Grb2 upregulation.

### Grb2 Controls Cardiomyocyte Mitochondrial Metabolism Through the Akt/mTOR Signaling Pathway

**FIGURE 5:**
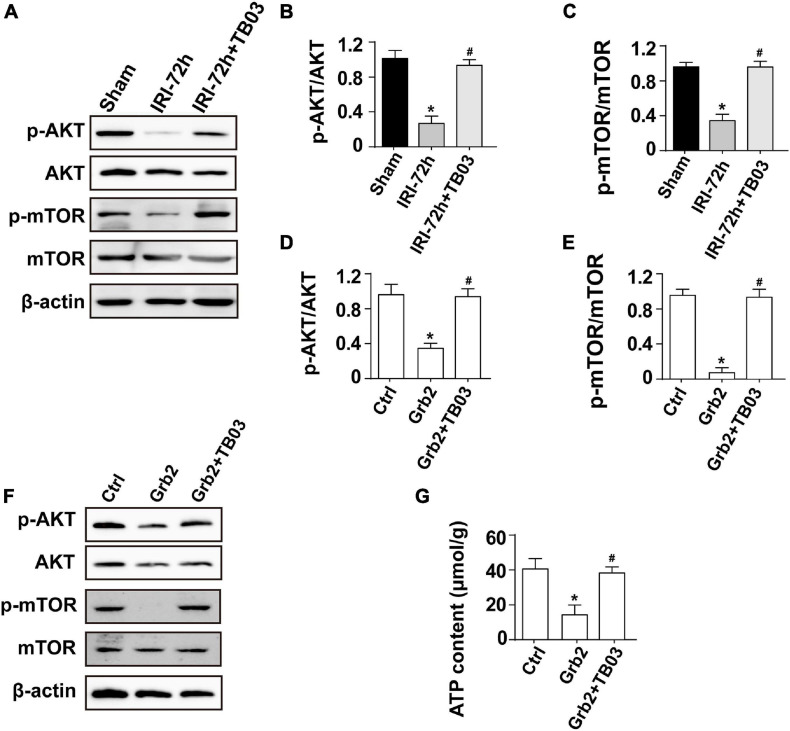
Grb2 activates the Akt/mTOR signaling pathway in cardiomyocytes. **(A–C)** Heart tissues from mice and the protein expression of p-Akt and p-mTOR were measured through western blots. **(D–F)** Cardiomyocytes were treated with exogenous Grb2 and its inhibitor TB03. Then, the protein expression of p-Akt and p-mTOR were measured through western blots. **(G)** The levels of intracellular ATP production were measured through Enhanced ATP Assay Kit. *p < 0.05 vs. sham group, ^#^*p* < 0.05 vs. IRI-72h group.

Three classical signaling pathways are under the control of Grb2 based on recent studies, including MAPK/ERK, MAPK/JNK, and PI3K/Akt (Zhang et al., 2003; Gui et al., 2012). Interestingly, Akt/mTOR pathway has been regarded as a key regulator of mitochondrial metabolism (Gan et al., 2016; Pan et al., 2018). Based on these findings, we asked whether Akt/mTOR pathway is the intracellular second messenger functioning downstream of Grb2 and thus affects mitochondrial metabolism in cardiomyocytes following AKI. To test our hypothesis, western blots were performed to analyze the alterations of Akt/mTOR pathway. As shown in Figures 5A–C, compared to the sham group, the expression of p-Akt was significantly downregulated in hearts from AKI-treated mice, an alteration that was followed by a decline in the expression of p-mTOR. Besides that, administration of TB03 significantly upregulated the levels of p-Akt and p-mTOR in hearts from AKI-treated mice. These data elucidate a possible link between Grb2 activation and Akt/mTOR pathway suppression in the context of AKI-mediated myocardial damage.

To better clarify the relationship between Grb2 and Akt/mTOR pathway, gain-of-function assay of Grb2 was performed in normal HL1 cardiomyocyte cell line using the recombinant mouse growth factor receptor-bound protein 2. Cardiomyocyte cultured with recombinant mouse growth factor receptor-bound protein 2 expressed less p-Akt and decreased p-mTOR when compared with the control group (Figures 5D–F). Furthermore, cardiomyocyte ATP production was also suppressed by recombinant mouse growth factor receptor-bound protein 2 (Figure 5G). In contrast, in TB03-pretreated cardiomyocyte, recombinant mouse growth factor receptor-bound protein 2 failed to repress the expression of p-Akt and p-mTOR (Figures 5D–F). Similarly, ATP production was also maintained in TB03-pretreated cardiomyocyte despite the administration of recombinant mouse growth factor receptor-bound protein 2 (Figure 5G). Overall, our results identify the Akt/mTOR pathway as one potential intracellular signal transducer of Grb2 upregulation in the pathogenesis of AKI-related cardiac damage.

### Circulating IL-6 Expression Is Increased in Response to AKI

**FIGURE 6:**
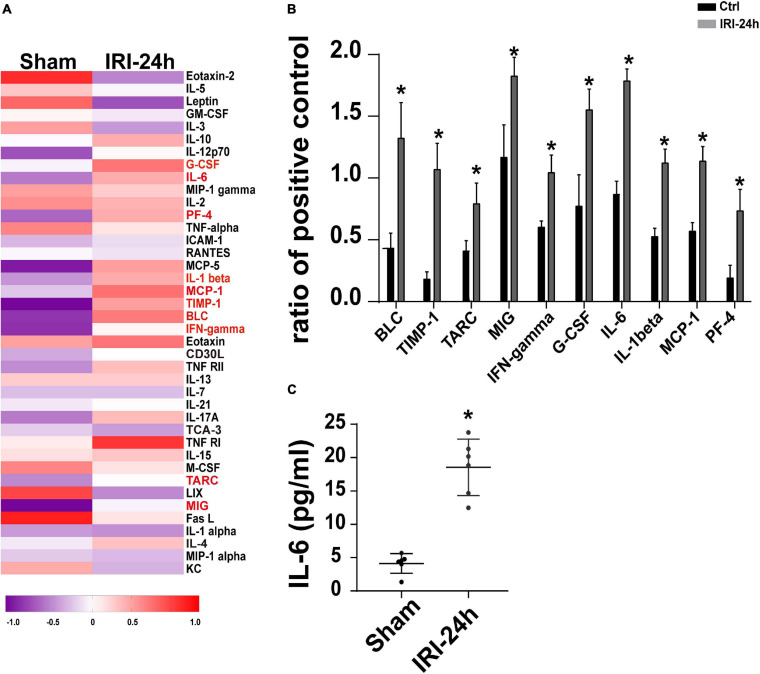
Circulating IL-6 expression is increased in response to acute kidney injury (AKI). **(A)** Heat map of altered serum cytokines differentially expressed in the heart 24 h after AKI. **(B)** The altered serum cytokines in 24 h after AKI. **(C)** ELISA was used to analyze the alterations of circulating IL-6 after AKI. *p < 0.05 vs. sham group.

The next experiments were performed to figure out the upstream regulator of Grb2 in AKI-mediated myocardial damage. Circulating inflammation cytokines have been reported to be the “connector” that is induced by AKI and acts as a contributor in triggering myocardial damage (Virzi et al., 2018; Clementi et al., 2019). Given that, we asked whether inflammation cytokines may be the upstream mediator of myocardial Grb2 upregulation after AKI. The Mouse Inflammation Array Q1 results demonstrate that serum cytokines were primarily altered at 24 h after AKI. Compared to the sham group, there were 10 cytokines significantly upregulated 24 h after AKI. The quantitative results demonstrated that IL-6, BLC, and TIMP-1 were the most pronounced cytokines upregulated by AKI (Figures 6A,B). Interestingly, IL-6 has been acknowledged as a critical inflammation parameter to evaluate the severity of AKI (Zhang et al., 2015; Andres-Hernando et al., 2017). More importantly, the causal role played by IL-6 in inducing Grb2 upregulation has been widely discussed (Lee et al., 1997; Bongartz et al., 2019). Therefore, AKI-mediated IL-6 may be a potential mechanism for Grb2 upregulation. In our study, ELISA assay further demonstrated that the concentration of IL-6 was up to approximately fourfold higher than those observed in the sham group (Figure 6C). Taken together, inflammation response, especially circulating IL-6 expression upregulation, may be an upstream signal for Grb2 upregulation.

### IL-6 Upregulates Grb2 Expression and Impairs Cardiomyocyte Bioenergetics

**FIGURE 7:**
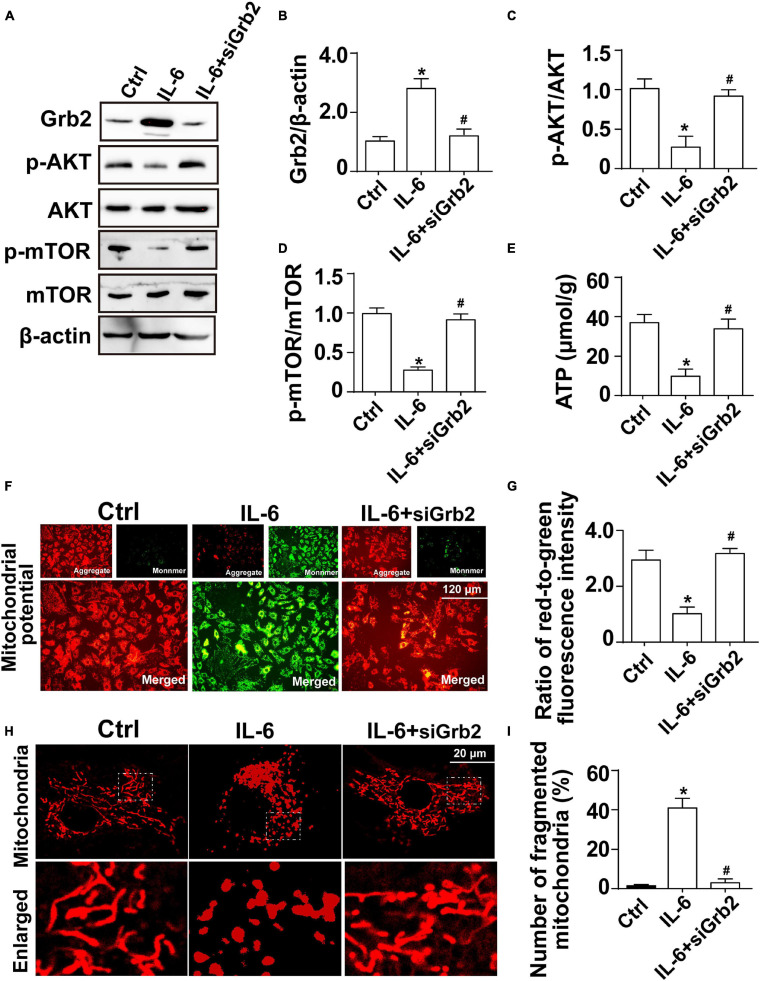
Exogenous IL-6 mediates cardiomyocyte mitochondrial damage and bioenergetics impairment through inhibition of the Akt/mTOR signaling pathway. **(A–D)** Cardiomyocytes were treated with exogenous IL-6. Then, the protein expression of Grb2, p-Akt, and p-mTOR, respectively, was measured through western blots. **(E)** The levels of intracellular ATP production were measured through Enhanced ATP Assay Kit. **(F,G)** Mitochondrial membrane potential was determined through JC-1 probe. The ratio of red-to-green fluorescence intensity was measured to reflect the alterations of mitochondrial membrane potential. **(H,I)** Immunofluorescence of mitochondria. Cardiomyocytes were treated with exogenous IL-6. *p < 0.05 vs. control group, ^#^*p* < 0.05 vs. IL-6 group.

**FIGURE 8:**
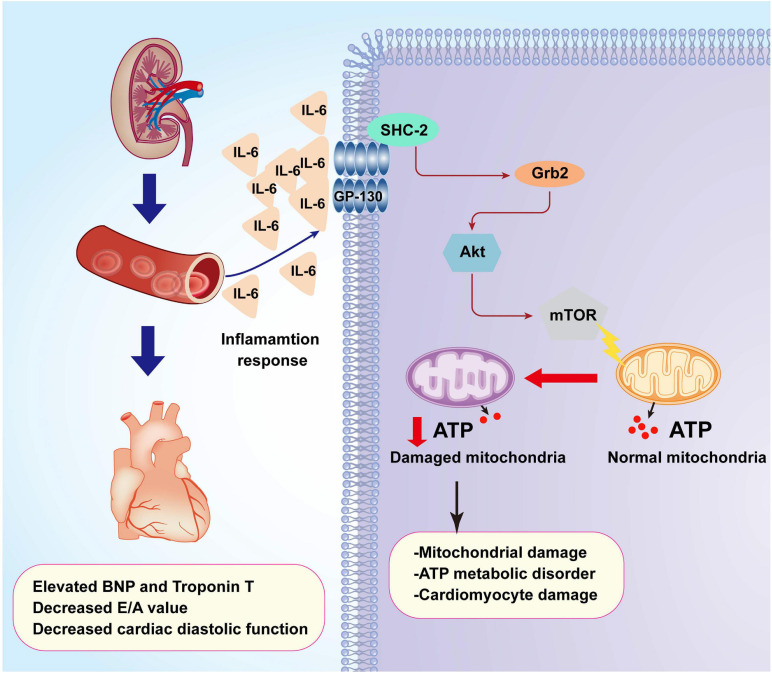
A summary of the pathological alterations of CRS-3. Acute kidney injury (AKI) promotes inflammation response which elevates the concentrations of IL-6. Increased IL-6 upregulates the expression of Grb2 which mediates cardiomyocyte metabolism disorder through affecting the Akt/mTOR signaling pathway. Dysregulated cardiomyocyte ATP metabolism impairs myocardial relaxation and exacerbates cardiac injury after AKI.

Further supporting the inflammation cytokine array findings, cardiomyocytes were treated with exogenous IL-6 to observe the alterations of Grb2, Akt/mTOR pathway, and cardiomyocyte bioenergetics. After administration of IL-6, the expression of Grb2 was significantly elevated in cardiomyocyte, an effect that was accompanied with a drop in the levels of p-Akt and p-mTOR (Figures 7A–D). Besides that, cardiomyocyte total ATP production was inhibited (Figure 7E), mitochondrial membrane potential was repressed (Figures 7F,G), and mitochondrial fission was activated in response to IL-6 treatment (Figures 7H,I). The above-mentioned data validated that IL-6 could be an upstream inducer of AKI-related cardiomyocyte damage through upregulation of Grb2, inhibition of Akt/mTOR pathway, and suppression of ATP production. Interestingly, in si-Grb2-pretreated cardiomyocyte, IL-6 supplementation failed to affect Grb2 expression, Akt/mTOR pathway activation, ATP production, and mitochondrial morphology. Taken together, the deleterious action of AKI on cardiac dysfunction is working through IL-6 upregulation-mediated Grb2 activation, Akt/mTOR pathway inhibition, and cardiomyocyte bioenergetics impairment (Figure 8).

## Discussion

CRS-3 is acute injury to the myocardium following AKI. Oxidative stress, inflammation response, hemodynamic disorder, cardiomyocyte apoptosis, and mitochondrial damage have been introduced to explain the pathogenesis underlying renal failure-induced cardiac damage (Rangaswami and Mathew, 2018; Ronco et al., 2018; Zannad and Rossignol, 2018). However, the intrinsic logical relationship among these pathological alterations has not been clarified, and the primary upstream regulator of these intracellular molecular biological events has not been identified. In our study, proteomic analysis illustrated that Grb2 may be a potential manager controlling heart function, cardiomyocyte viability, cellular bioenergetics, and mitochondrial function. Blockade of Grb2 after AKI significantly attenuates cardiac damage and sustains myocardial functions through a mechanism involving the re-activation of Akt/mTOR pathway and preservation of cardiomyocyte bioenergetics. Besides that, inflammation cytokine array further identifies IL-6 as an upstream signal for myocardial Grb2 activation. In cardiomyocytes treated with Grb2 inhibitor, exogenous supplementation of IL-6 failed to induce Grb2 upregulation, suppress Akt/mTOR pathway, and disrupt cardiomyocyte bioenergetics. These findings explain the molecular alterations in myocardium after AKI and provide a promising target to mitigate cardiac dysfunction induced by CRS-3.

In our results, we found that cardiac diastolic function rather than systolic function was impaired by AKI. This finding is similar to previous observations (Sumida et al., 2015). Three mechanisms could be used to explain this finding. First, mild cardiomyocyte apoptosis occurs in response to AKI; this pathological alteration is in contrast to myocardial infarction and cardiac ischemia–reperfusion injury which are featured by ∼50% of cardiomyocyte apoptosis (Shen et al., 2018). Due to a strong myocardial compensatory capacity, the slight loss of functional cardiomyocytes is not followed by a significant decrease in systolic function (Zhou et al., 2018a; Wang et al., 2020a). Second, inflammation response was elevated in myocardium following AKI, which contributes to myocardial edema (Kelly, 2003). This alteration limits myocardial compliance and thus disturbs cardiac relaxation. Third, we noted mitochondrial damage in cardiomyocytes following AKI, which may reduce the energy supply for the myocardium. Compared to systole, cardiomyocytes consume more ATP at the stage of diastole (Bacmeister et al., 2019; Bøtker, 2019). Therefore, diastolic function rather than systolic property is firstly lessened in response to mitochondrial damage. In addition, cardiac coronary circulation determines the fresh blood to the myocardium, and therefore more studies are required to explore whether cardiac microvascular damage is triggered by AKI and contributes to cardiac dysfunction.

Over the past decades, there are many studies that explore the alterations of Grb2, especially in renal cell cancer, podocyte fibrosis, cardiac hypertrophy, and myocardial remodeling (Mohanty and Bhatnagar, 2018; Chang et al., 2019; Sun et al., 2019) as we have introduced earlier. Therefore, the regulatory effects of Grb2 on cell apoptosis, inflammation response, and mitochondrial damage have been established (Chang et al., 2019). The novel finding of our study is that we performed a non-bias proteomic analysis which screens out Grb2 as a primary factor involved in myocardial damage following AKI. Increased Grb2 inhibited the activity of the Akt/mTOR pathway, resulting in damage to mitochondrial bioenergetics with a drop in the content of intracellular total ATP production. Due to limited ATP production, cardiomyocyte relaxation is disrupted, and heart function is compromised. There is another result that should be pointed out. Based on our proteomic analysis, the expression of Grb2 in the kidney (unpublished data) was increased 24 h after AKI and decreased to physiological levels 72 h after AKI. In contrast, the expression of Grb2 was progressively increased in the myocardium from 24 to 72 h after AKI. There is one explanation for this discrepancy between the kidney and the heart. The kidney may be provoked and/or attacked before cardiac damage, although this speculation lacks enough data to be supported.

To understand the upstream regulator mechanism underlying myocardial Grb2 upregulation following AKI, inflammation cytokine array was performed, and the results uncovered that IL-6 would be a potential mediator triggering AKI-related cardiac damage. This finding is consistent with those of previous studies (Funahashi et al., 2020; Ghionzoli et al., 2020). During AKI, ischemic injury triggers non-specific adaptive immunity pathways with consequent activation and recruitment of inflammatory cells in the kidneys (Di Lullo et al., 2019). This alteration is accompanied with an increase in systemic inflammation response which is manifested by increased levels of circulating pro-inflammatory cytokines such as TNFα, IL-1, and IL-6 (Funahashi et al., 2020; Ghionzoli et al., 2020). Excessive inflammation injury induces cardiomyocyte dysfunction through various mechanisms including, but not limited to, the induction of cardiomyocyte apoptosis, promotion of intracellular energy depletion, stimulation of cardiomyocyte oxidative stress, and disruption of myocardial microcirculation (Lee et al., 2018; Ronco et al., 2018; Di Lullo et al., 2019). IL-6 has been reported to be an important mediator of cardiac inflammation after AKI (Panico et al., 2019), although the pathological role of IL-6 in cardiac dysfunction following AKI has not been fully understood. In the present study, our data showed that increased IL-6 induced an upregulation of Grb2, resulting into the impairment of mitochondrial bioenergetics. In fact, the relationship between IL-6 upregulation and Grb2 activation has been reported in human myeloma (Berger and Hawley, 1997) and lymphoma (Giordano et al., 1997). However, it remains unknown whether circulating IL-6 upregulation is a direct result or is a secondary consequence of AKI.

Lastly, both our data and those of previous studies verify that mitochondria, especially mitochondria-related cardiomyocyte bioenergetics, are the key targets regulating AKI-related cardiac dysfunction (Husain-Syed et al., 2020). A previous finding described (Sumida et al., 2015) that mitochondrial fission, possibly controlled by Drp1, is activated in response to AKI, resulting in mitochondrial bioenergetics reduction. A recent study also reported that mitochondrial calcium overload happens as a result of AKI, leading to mitochondrial potential dissipation and ATP deficiency. Our data reconfirmed that mitochondrial fission was enhanced by AKI, which was followed by mitochondrial fragmentations. Abnormal mitochondrial morphology shift may hinder mitochondrial oxidative phosphorylation, resulting in mitochondrial membrane potential reduction and intracellular ATP depletion. These findings will extend the pathogenic role played by mitochondrial bioenergetics in CRS-3. However, an open question is how Grb2 affects mitochondrial fission in cardiomyocytes following AKI.

Our results altogether identify that CRS-3 is caused by IL-6/Grb2 upregulations which contribute to cardiac dysfunction through inhibiting the Akt/mTOR signaling pathway and inducing cardiomyocyte mitochondrial bioenergetics impairment. This finding opens a new window to understand the pathophysiological mechanism underlying CRS-3.

There are several limitations in our study. Firstly, although we established a mouse model of CRS-3, the clinical pathological alterations of CRS-3 are relatively complex because many patients with CRS-3 are often diagnosed with diabetes, hyperlipidemia, hypertension, or myocardial infarction. It requires more experiments to figure out whether these complications have a contributory role in the pathogenesis of CRS-3. Secondly, our data demonstrate that IL-6 and Grb2 could be the potential regulators during CRS-3. However, this notion should be further verified in patients with CRS-3 using clinical samples. Thirdly, although pathway blockers or inhibitors are used in our study to perform functional studies, genetically modified mouse will provide more solid data in a further research.

## Data Availability Statement

The raw data supporting the conclusions of this article will be made available by the authors, without undue reservation.

## Ethics Statement

The animal study was reviewed and approved by the Laboratory Animal Center, Chinese PLA General Hospital.

## Author Contributions

XS, GC, and XC designed the experiments. JW, XW, and JL performed the experiments. SC and RL acquired and analyzed the data. BF, MG, and CW wrote the manuscript. YS and QC analyzed and discussed the data. All the authors approved the submitted manuscript.

## Conflict of Interest

The authors declare that the research was conducted in the absence of any commercial or financial relationships that could be construed as a potential conflict of interest.
